# Comparative transcriptome analysis of *Rimicaris* sp. reveals novel molecular features associated with survival in deep-sea hydrothermal vent

**DOI:** 10.1038/s41598-017-02073-9

**Published:** 2017-05-17

**Authors:** Jian Zhang, Qing-lei Sun, Zhen-dong Luan, Chao Lian, Li Sun

**Affiliations:** 1 Key Laboratory of Experimental Marine Biology, Institute of Oceanology, Chinese Academy of Sciences, Qingdao, 266071 China; 2 Laboratory for Marine Biology and Biotechnology, Qingdao National Laboratory for Marine Science and Technology, Qingdao, China; 3 Key Laboratory of Marine Geology and Environment, Institute of Oceanology, Chinese Academy of Sciences, Qingdao, 266071 China; 4 Deep Sea Research Center, Institute of Oceanology, Chinese Academy of Sciences, Qingdao, 266071 China

## Abstract

Shrimp of the family Alvinocarididae are the predominant megafauna of deep-sea hydrothermal vents. However, genome information on this family is currently unavailable. In the present study, by employing Illumina sequencing, we performed the first *de novo* transcriptome analysis of the gills of the shrimp *Rimicaris* sp. from the hydrothermal vent in Desmos, Manus Basin. The analysis was conducted in a comparative manner with the shrimp taken directly from the vent (GR samples) and the shrimp that had been maintained for ten days under normal laboratory condition (mGR samples). Among the 128,938 unigenes identified, a large number of differentially expressed genes (DEGs) between the GR and mGR samples were detected, including 2365 and 1607 genes significantly upregulated and downregulated, respectively, in GR. The DEGs covered diverse functional categories. Most of the DEGs associated with immunity were downregulated in GR, while most of the DEGs associated with sulfur metabolism and detoxification were upregulated in GR. These results provide the first comprehensive transcriptomic resource for hydrothermal vent *Rimicaris* and revealed varied categories of genes likely involved in deep-sea survival.

## Introduction

Deep-sea hydrothermal vents are unique ecosystems that are sporadically distributed along mid-oceanic ridges, back-arc basins, volcanic arcs, and active seamounts^1^. Such ecosystems are sustained by the primary production of chemolithoautotrophic microorganisms, which support highly diverse and dense fauna communities^2^. The extreme conditions in hydrothermal vents, such as sulfide, heavy metals, high pressure, and low dissolved oxygen, pose severe challenges to local inhabitants^3^. It is unclear how the living organisms in hydrothermal vents adapt to and survive the harsh environments.

Sustained exposure to high levels of hydrogen sulfide (H_2_S) can limit the ability of the organism to survive and reproduce^4^. The primary cytotoxic effect of H_2_S is the interruption of the mitochondrial respiratory chain by directly inhibiting cytochrome c oxidase^5^. H_2_S can also harm organisms by disrupting calcium homeostasis and reducing the affinity of oxygen transport proteins^6^, thereby causing oxidative damage to DNA and RNA^7, 8^. Organisms colonizing H_2_S-rich habitats have evolved a variety of strategies to cope with the continuous exposure to this toxicant. These strategies include exclusion approaches, modifications of toxicity targets, increased capacity for detoxification, and symbioses with sulfur-oxidizing microbes^9^. For organisms living in deep-sea hydrothermal systems, the physiological and genetic mechanisms underlying H_2_S adaptation are poorly understood. Previous studies have shown that in the hydrothermal vent-associated giant tube worm *Riftia pachyptila* and clam *Solemya velum*, hemoglobin delivers H_2_S to endosymbiotic bacteria^10, 11^, and that in the mussel *Bathymodiolus platifrons* from cold seeps, several key enzymes, such as sulfide:quinone oxidoreductase (SQR) and sulfur dioxygenase (SDO), participate in sulfide detoxification^12^.

Shrimps of the family Alvinocarididae inhabit the Atlantic, Pacific, and Indian Oceans; they comprise the predominant faunal biomass of various hydrothermal ecosystems, in some cases even reaching an abundance of thousands of individuals per square meter^13^. Within this family, the genus *Rimicaris* is one of the most studied hydrothermal crustaceans. Currently, three species of this genus have been described, i.e., *Rimicaris exoculata*, *Rimicaris hybisae*, and *Rimicaris kairei* ^14–16^. *R*. *exoculata* is the predominant species of most hydrothermal vents along the Mid-Atlantic ocean ridge^17^ and has also been discovered in other vent fields such as that in the Mid-Cayman Spreading Center and southwest Indian Ocean ridge^14, 15^. To date, no information on the genome and transcriptome of deep-sea shrimp is available, and the mechanism underlying their environmental adaptation and survival is unclear.

In a recent scientific cruise, we had obtained shrimp samples from a hydrothermal vent in Desmos, Manus Basin. Given the extreme hydrothermal condition, such as a high centration of H_2_S, we hypothesized that the local animals very likely exhibited a distinct gene expression pattern essential to survival in the habitat. In order to find out the hydrothermal vent-associated transcription profile in these shrimp, we employed the Illumina Hiseq 4000 platform and conducted, in a comparative manner, high-throughput transcriptome analysis of the shrimp taken directly from the vent site and the shrimp that had been maintained under normal laboratory condition. By searching for differentially expressed genes under these two conditions, we hoped to find genes potentially contributing to the survival of the shrimp in hydrothermal systems, thus providing the first insights and clues to the survival strategy and environmental adaptation mechanism of deep-sea shrimp.

## Results

### Phylogenetic analysis of shrimp

**Figure 1:**
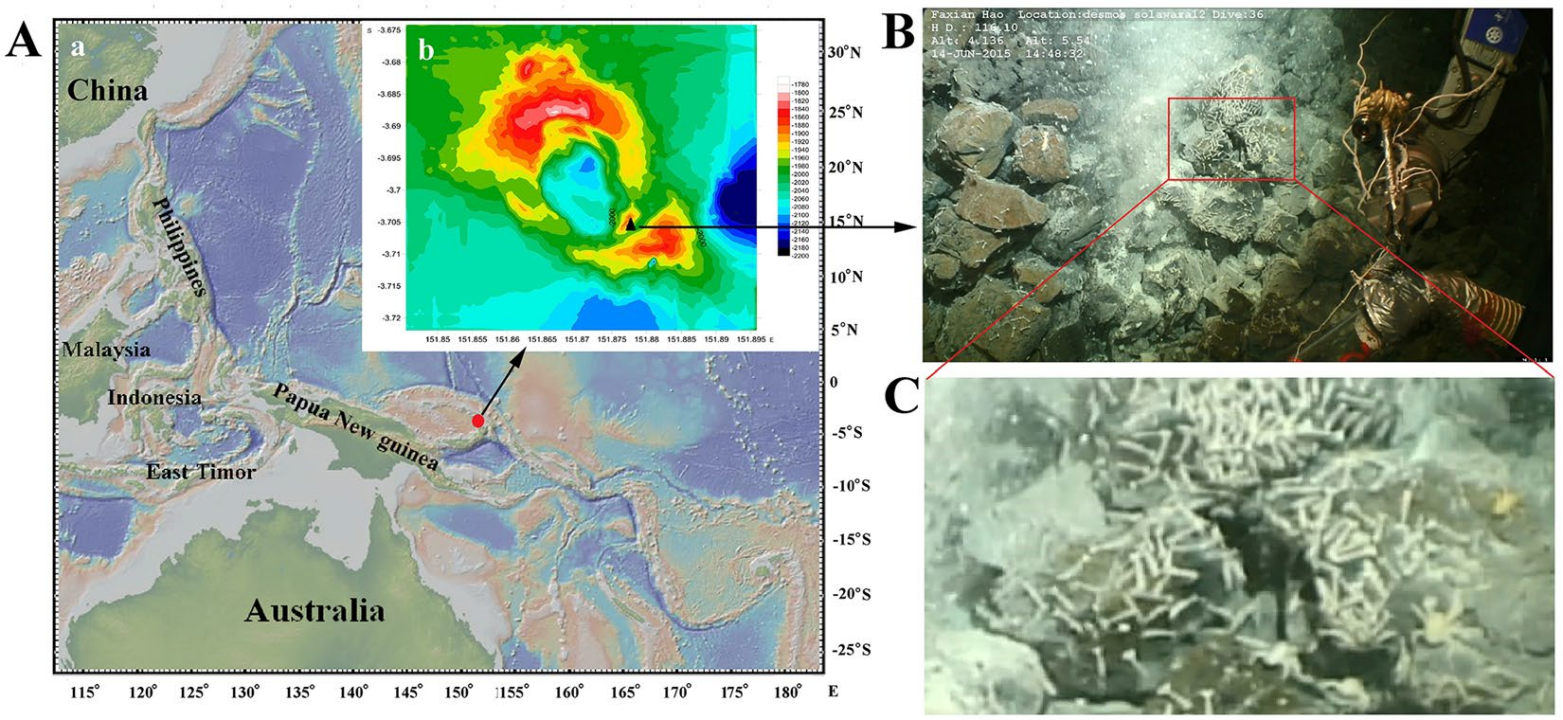
Location of the sampling site. (A) Location map of Desmos Manus basin (marked with “”) and the newly discovered hydrothermal site in the basin (marked with “” in Ab). Aa was generated with GeoMapApp version 3.6.0 (http://www.geomapapp.org) using Global Multi-Resolution Topography synthesis database, and Ab was generated using Surfer (Golden Software Inc., ver. 12.0) with the data obtained by the scientific research vessel “KEXUE” during the cruise reported in this study. (**B**) Hydrothermal vent and the associated shrimp communities. (**C**) Enlarged image of the boxed region in (**B**).

**Figure 2:**
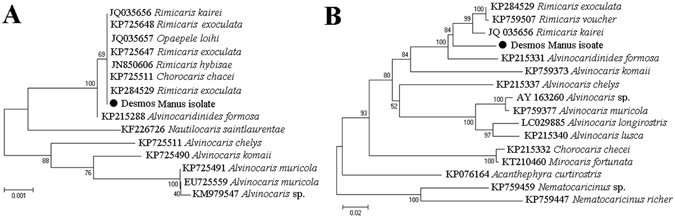
Phylogenetic analysis based on 16S rRNA and COI genes. The nucleotide sequences of 16S rRNA (**A**) and COI (**B**) genes of the Desmos Manus isolate were compared with those of the shrimp that were available in GenBank. Phylogenetic trees were generated by distance-based neighbor-joining method with 1,000 bootstrap replicates. The sequences marked with “●” are from this study.

The shrimp sampling site was described in Materials and Methods and shown in Fig. 1. Sequence analysis showed that the shrimp examined in the present study (Desmos Manus isolate) shares the highest 16S rRNA sequence identity (100%) with *R*. *kairei* and the highest COI sequence identity (95.7%) with *R*. *exoculata*. Phylogenetic reconstruction based on the 16S rRNA gene clustered the Desmos Manus isolate with the genera of *Rimicaris*, *Opaepele*, and *Chorocaris* of Alvinocarididae, and separated this cluster from the genera of *Alvinocaris* and *Nautilocaris* of Alvinocarididae (Fig. 2A). Phylogenetic analysis of the COI gene clustered the Desmos Manus isolate with the genus of *Rimicaris*, and separated this cluster from the other genera of Alvinocarididae (Fig. 2B). These results indicated that the Desmos Manus isolate was a species of the genus *Rimicaris* and thus was referred to as *Rimicaris* sp.

### Transcriptome sequencing and data assembly

A total of 134,297,376 raw reads were generated from the three gill samples that were immediately isolated from the shrimp on board (GR1 to GR3), and a total of 147,057,894 raw reads were generated from the three gill samples of the shrimp that that been maintained in the laboratory (mGR1 to mGR3) (Table 1). The raw sequencing data from GR and mGR were deposited to the NCBI sequence read archive database under the accession numbers SRR4342052 and SRR4342053, respectively. After removing adaptor sequences, ambiguous nucleotides, and low-quality sequences, 19.38G and 21.41G clean bases were obtained for the GR and mGR samples, respectively, with a GC average percentage of 38.1% and over 97.10% reads in all six samples exceeding Q20 (Table 1), which indicated a high quality of sequencing. Clean reads were assembled into 599,502 transcripts with an N50 length of 650 bp and an average length of 517 bp (Fig. S1). The length range (201 bp to 20,436 bp) and distribution of all transcripts are shown in Fig. S1.

**Table 1:** Quality of sequencing.

Sample	Raw reads	Clean reads	Clean bases (G)	Q20 (%)	Error (%)	GC (%)
GR1	45,335,746	44,117,636	6.62	97.15	0.01	39.67
GR2	43,032,318	40,732,820	6.11	97.29	0.01	37.21
GR3	45,929,312	44,320,664	6.65	97.14	0.01	39.86
**Total**/**Average**	134,297,376^a^	129,171,120^a^	19.38^a^	97.19^b^	0.01^b^	38.91^b^
mGR1	56,394,568	54,726,416	8.21	97.12	0.01	37.52
mGR2	40,354,318	39,172,356	5.88	97.10	0.01	37.11
mGR3	50,309,008	48,816,890	7.32	96.94	0.01	37.43
**Total**/**Average**	147,057,894^a^	142,715,662^a^	21.41^a^	97.05^b^	0.01^b^	37.35^b^

^a^Total number of data from the three samples.

^b^Average number of data from the three samples.

### Functional annotation of the transcripts

To estimate the function of the transcripts, the longest transcript of each gene was selected and designated as unigene, which was then subjected to further BLAST analysis using public databases. A total of 128,938 (26.38%) unigenes had significant hits in the NCBI Nr, Nt, SwissProt, KOG, or KEGG database (Table 2). Specifically, there were 69,635 unigenes (14.24%) showing significant BLAST hits against known sequences in the Nr database (Table 2); the best hit of the majority of the annotated unigenes and the *E*-value distributions of the matched sequences are shown in Fig. S2.

**Table 2:** Summary of unigene annotation.

Database	Number of annotated unigenes	Percentage of annotated genes	Unique hits
Nr	69,635	14.24%	8,802
Nt	26,320	5.38%	4,528
KEGG Ortholog (KO)	35,696	7.3%	205
SwissProt	60,976	12.47%	1,014
PFAM	96,911	19.82%	95
GO	98,192	20.09%	223
KOG	42,725	8.74%	8,802
Annotated in all databases	7,627	1.56%	\
Annotated in at least one database	128,938	26.38%	\

### DEGs between GR and mGR

**Figure 3:**
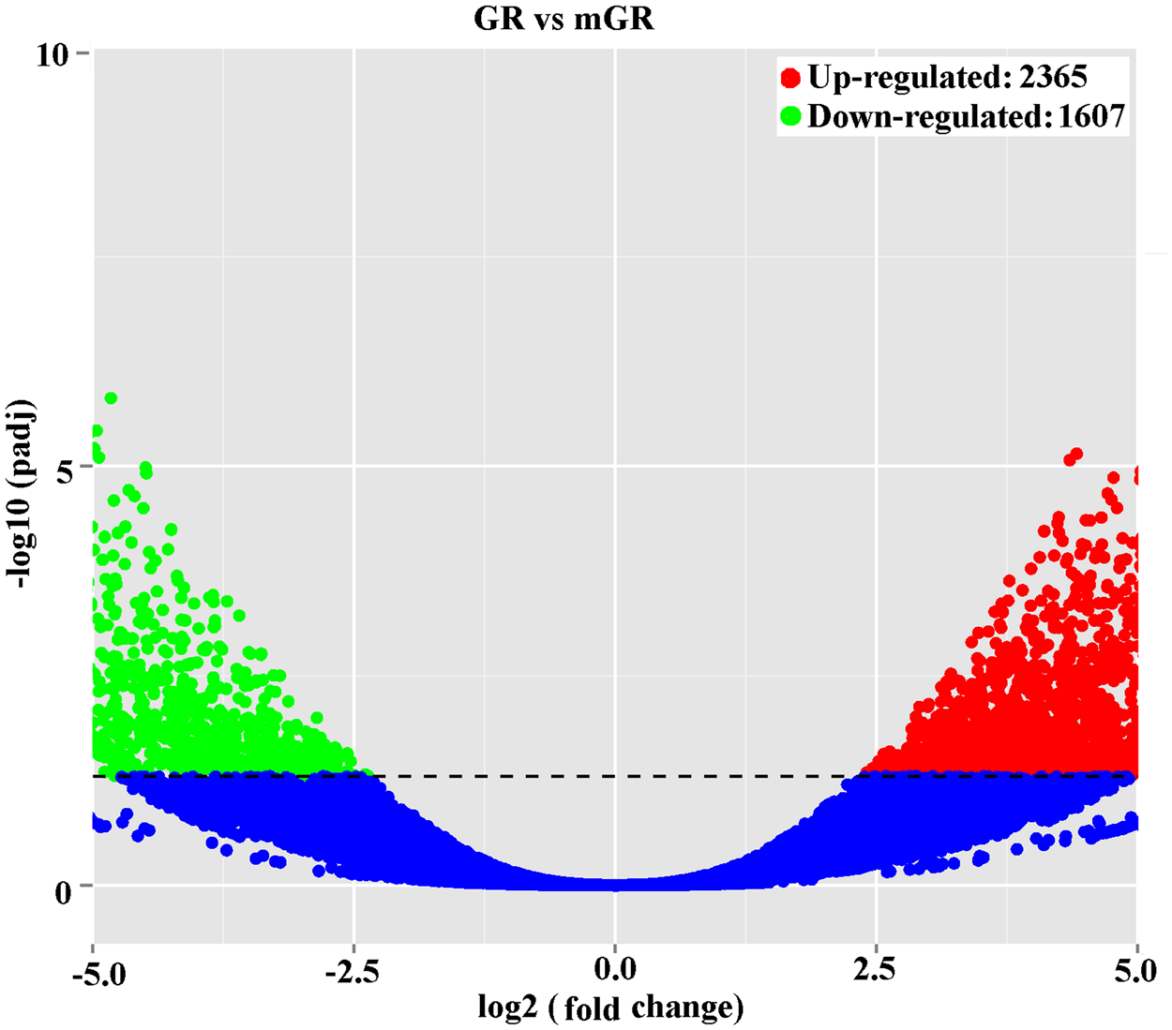
Volcano plot displaying differential expressed genes (DEGs) between GR and mGR samples. The red and green dots represent upregulated and downregulated DEGs, respectively, in the GR samples; the blue dots represent non-DEGs. A total of 3,972 unigenes were identified as differentially expressed (padj < 0.05).

**Figure 4:**
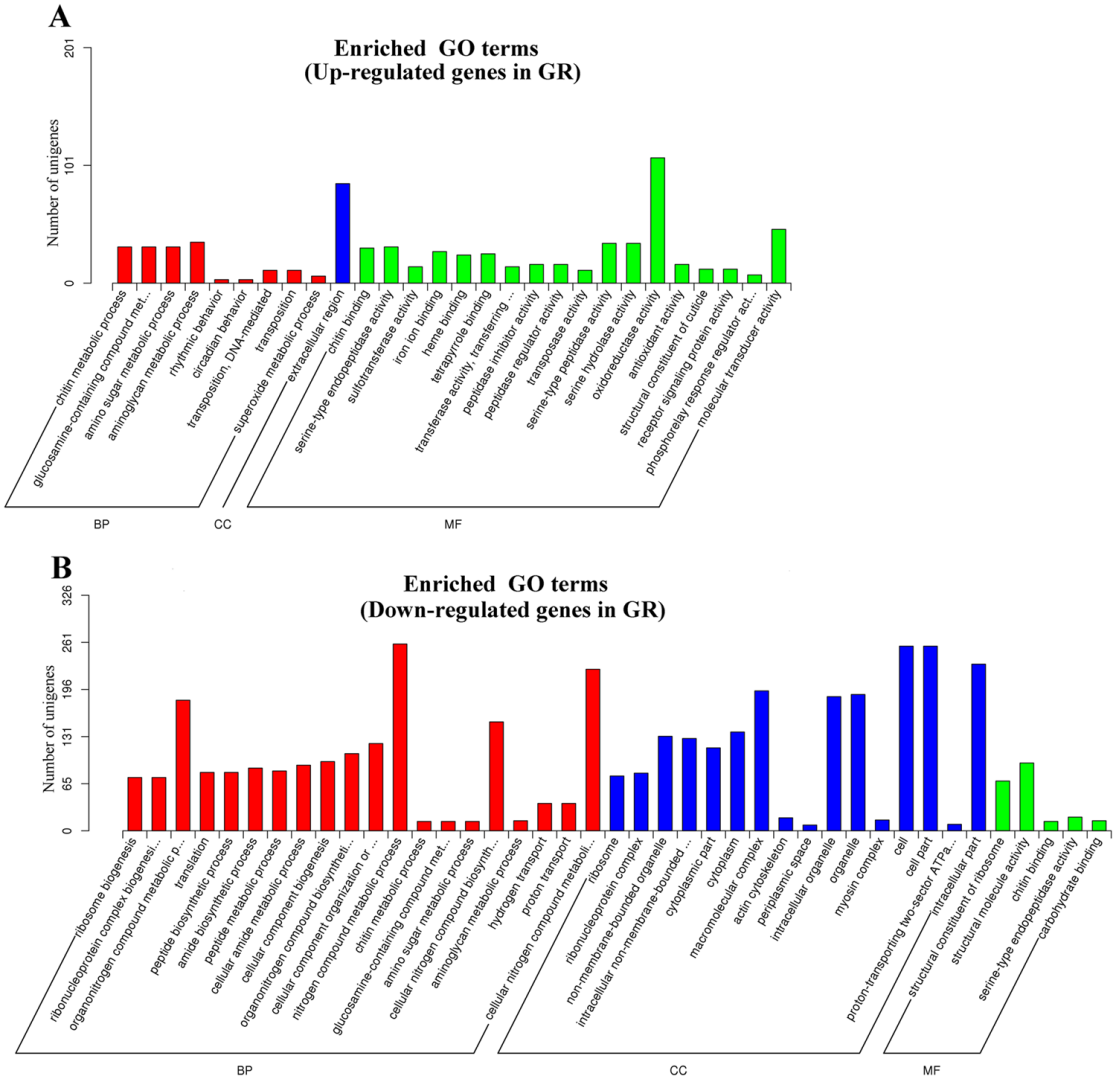
GO enrichment analysis of differential expressed genes (DEGs) compared to the whole transcriptome background. Upregulated DEGs (**A**) and downregulated DEGs (**B**) in GR samples with GO IDs that were categorized into three main categories shown in different colors: biological process (BP, red), cellular component (CC, blue), and molecular function (MF, green).

Compared to the mGR samples, a total of 3,972 unigenes were identified as DEGs in the GR samples, with 2,365 (59.51%) genes showing higher expression levels in GR than in mGR and 1,607 (40.49%) genes showing lower expression levels in GR than in mRG (Fig. 3). For functional annotation and classification, all DEGs were subjected to GO enrichment analysis. Of the 3972 DEGs, 1006 upregulated genes and 653 downregulated genes had GO ID and were categorized (Fig. 4). In the category of ‘molecular function’, 18 upregulated subcategories were significantly enriched, among which three subcategories potentially involved in environmental adaptation/survival were detected, i.e., sulfotransferase activity, antioxidant activity, and transposase activity (Fig. 4A). In the category of ‘cellular component’, the upregulated subcategory of ‘extracellular region’ was significantly enriched (Fig. 4A), within this subcategory were the DGEs of chitin binding peritrophin, cuticular protein, obstructor B, gastrolith protein, and laminin (Table 3), which are involved in the synthesis of calcified cuticular structures in invertebrates that may act as barriers to prevent the entry of H_2_S into the body^9, 18^.

**Table 3:** Summary of DEGs involved in sulfur metabolism, antioxidation and detoxification, and immunity.

Transcript ID	Expression level		Log2 fold change	padj^a^	Transcript length	Accession no.	Nr E-Value	Nr annotation
	GR	mGR						
**Sulfur metabolism**								
Sulfide:quinone oxidoreductase								
c52828_g1	45.2	17.4	2.3	4.35E-02	204	ACO13065.1	8.28E-27	Sulfide:quinone oxidoreductase [*Lepeophtheirus salmonis*]
Rhodanese domain protein								
c235883_g1	513.3	82.6	2.6	1.42E-02	414	EJY73421.1	1.18E-07	Rhodanese domain protein [*Oxytricha trifallax*]
c317563_g2	29.3	3.1	3.2	3.20E-03	246	XP_006626376.1	3.06E-14	Thiosulfate sulfurtransferase/rhodanese-like [*Lepisosteus oculatus*]
c61012_g1	7.3	36.1	−2.3	2.35E-02	597	XP_005966937.1	7.08E-46	Thiosulfate/3-mercaptopyruvate sulfurtransferase 1 [*Pantholops hodgsonii*]
c331935_g1	4.5	23.8	−2.4	5.20E-03	594	XP_005966937.1	2.55E-46	Thiosulfate/3-mercaptopyruvate sulfurtransferase 1 [*Pantholops hodgsonii*]
Sulfite oxidase								
c318457_g1	652.1	62.9	3.2	3.32E-02	578	XP_011644963.1	7.87E-56	Probable sulfite oxidase, mitochondrial [*Pogonomyrmex barbatus*]
c320892_g1	1651.0	296.5	2.5	3.20E-03	1698	KFM61096.1	0	Sulfite oxidase, mitochondrial, partial [*Stegodyphus mimosarum*]
Sulfotransferase								
c319825_g3	605.3	46.2	3.5	3.55E-02	3060	XP_012259172.1	9.87E-130	Heparan sulfate 2-O-sulfotransferase pipe isoform X3 [*Athalia rosae*]
c312613_g1	36.1	1.1	4.5	3.33E-03	1602	XP_001950548.2	8.99E-67	Heparan sulfate 2-O-sulfotransferase pipe [*Acyrthosiphon pisum*]
c294043_g1	21.2	0.7	4.6	2.18E-02	1505	KFM76191.1	9.34E-65	Galactosylceramide sulfotransferase, partial [*Stegodyphus mimosarum*]
c310298_g1	1496.8	44.1	4.8	1.63E-04	1299	AJC52502.1	1.05E-91	Estrogen sulfotransferase [*Scylla olivacea*]
c32047_g1	27.5	0.2	5.9	2.37E-03	1318	AJC52502.1	5.24E-58	Estrogen sulfotransferase [*Scylla olivacea*]
c275050_g1	527.1	33.2	3.8	6.98E-03	1016	XP_001842581.1	3.05E-18	Chondroitin 4-sulfotransferase [*Culex quinquefasciatus*]
c321100_g1	416.3	23.7	3.9	3.28E-03	3271	LRGB01002451.1	1.12E-78	Carbohydrate sulfotransferase 5 [*Daphnia magna*]
c297891_g2	350.8	2.2	6.7	1.10E-05	1651	XP_008204389.1	1.74E-54	Carbohydrate sulfotransferase 11-like [*Nasonia vitripennis*]
c321023_g2	185.8	1.4	6.6	4.58E-06	1971	XP_011315535.1	2.57E-32	Carbohydrate sulfotransferase 11-like [*Fopius arisanus*]
c309372_g1	3367.2	23.2	6.8	3.81E-09	2192	KDR14955.1	3.56E-37	Carbohydrate sulfotransferase 11, partial [*Zootermopsis nev*a*densis*]
c281591_g1	263.0	6.8	4.9	1.95E-03	1953	KDR20510.1	2.61E-31	Carbohydrate sulfotransferase 11 [*Zootermopsis nevadensis*]
c289839_g1	930.2	48.2	4.0	3.88E-03	1185	KDR22013.1	3.18E-16	Carbohydrate sulfotransferase 11 [*Zootermopsis nevadensis*]
c304186_g1	666.0	28.7	4.2	7.91E-03	1966	KK853243.1	3.98E-75	Carbohydrate sulfotransferase 11 [*Zootermopsis nevadensis*]
**Antioxidation and detoxification**								
Glutathione peroxidase								
c319517_g1	49538.8	5728.6	3.0	2.20E-02	1407	AET43964.1	5.57E-63	Glutathione peroxidase, partial [*Reishia clavigera*]
Superoxide dismutase								
c277048_g4	31.9	1.4	4.3	9.00E-03	1821	BAP28201.1	1.98E-77	Copper/zinc superoxide dismutase isoform 2 [*Marsupenaeus japonicus*]
c287566_g1	135.2	3.6	4.8	1.64E-03	1274	BAP28204.1	6.00E-59	Copper/zinc superoxide dismutase isoform 5 [*Marsupenaeus japonicus*]
c518684_g1	0	11.3	−5.8	4.06E-02	589	AFK10936.1	1.63E-48	Superoxide dismutase [*Callorhinchus milii*]
c293292_g1	3.9	104.69	−4.4	9.19E-03	1086	AAZ29240.1	3.56E-46	Copper/zinc superoxide dismutase [*Macrobrachium rosenbergii*]
Cytochrome P450								
c322077_g1	69.6	8.2	3.0	3.54E-02	946	ADD63783.1	3.90E-98	Cytochrome P450 [*Litopenaeus vannamei*]
c262085_g1	205.0	5.9	4.8	1.62E-03	1703	AIY69132.1	0	Cytochrome P450 [*Neocaridina denticulata*]
c297585_g1	244.8	14.7	3.9	4.28E-03	2040	AFA26603.1	0	Cytochrome P450 V20 [*Macrobrachium nipponense*]
c243124_g2	18927.2	119.1	6.8	3.52E-06	1676	XP_002425178.1	1.62E-101	Chitin binding peritrophin-A, putative [*Pediculus humanus corporis*]
c303676_g1	1504.8	32.3	5.1	3.57E-03	423	ACB05778.1	2.00E-33	Cuticular protein [*Artemia franciscana*]
c36214_g1	8411.6	57.5	6.8	1.41E-07	1095	AJZ68821.1	3.34E-87	Obstructor B [*Locusta migratoria*]
c301020_g1	26403	2100.7	3.5	3.51E-03	2083	ACC97407.1	0	Gastrolith protein [*Cherax quadricarinatus*]
c322133_g1	1194.4	127.5	3.1	1.96E-02	5514	KDR15871.1	0	Laminin subunit beta-1 [*Zootermopsis nevadensis*]
c325690_g1	1227.9	7.4	6.8	6.60E-06	429	ACB05778.1	3.44E-32	Cuticular protein [*Artemia franciscana*]
c302587_g1	526.8	0.7	6.7	1.72E-02	405	ACB05778.1	3.63E-43	cuticular protein [*Artemia franciscana*]
c122761_g1	14684.3	337.8	5.2	5.04E-06	900	AGG20312.1	4.30E-142	Peritrophin [*Palaemon carinicauda*]
c324109_g1	866.9	110.6	2.9	9.71E-03	9531	KDR22194.1	0	Laminin subunit alpha-1 [*Zootermopsis nevadensis*]
**Immunity**								
Lectin								
c283074_g1	2.7	148.4	−5.4	5.98E-05	948	AEH05998.1	6.71E-83	C type lectin containing domain protein [*Litopenaeus vannamei*]
c316369_g1	4.5	93.1	−4.1	2.37E-03	585	AAZ29608.1	6.48E-09	C-type lectin [*Penaeus monodon*]
c264506_g1	0.3	29.3	−5.5	1.37E-02	696	AGL46986.1	1.14E-42	C-type lectin [*Procambarus clarkii*]
c312469_g4	5.7	65.2	−3.3	3.19E-02	1056	AGZ95685.1	7.35E-113	C-type lectin 1 [*Palaemon modestus*]
c64342_g1	2797.6	252.6	3.3	1.06E-02	750	ACJ06432.1	1.08E-116	C-type lectin 4 [*Fenneropenaeus chinensis*]
c288904_g1	111.6	0.5	7.0	2.97E-04	549	AAX63905.1	2.39E-09	C-type lectin protein [*Fenneropenaeus chinensis*]
c315345_g1	7.3	129.2	−3.9	2.23E-02	1086	ACC86854.1	6.07E-24	C-type lectin-like [*Portunus trituberculatus*]
c292643_g1	5.1	113.5	−4.2	1.70E-02	612	ACC86854.1	4.94E-45	C-type lectin-like [*Portunus trituberculatus*]
c304905_g1	0.3	197.5	−8.1	1.71E-06	540	ACC86854.1	2.38E-31	C-type lectin-like [*Portunus trituberculatus*]
c304072_g1	2.5	89.2	−4.9	2.20E-04	549	XP_007570651.1	3.12E-06	C-type lectin lectoxin-Phi1-like, partial [*Poecilia formosa*]
Antimicrobial peptide								
c276042_g1	1.5	51.3	−4.8	1.85E-03	345	AFN80341.1	1.28E-12	Antimicrobial peptide type 1 precursor [*Pandalopsis japonica*]
c292762_g1	153.7	1909.3	−3.5	1.64E-03	450	AFN80342.1	2.55E-36	Antimicrobial peptide type 1 precursor [*Pandalopsis japonica*]
c273208_g2	0	84.5	−8.3	1.11E-04	323	AGU01545.1	2.82E-19	Antimicrobial peptide type 2 precursor IIc [*Pandalopsis japonica*]
Anti-lipopolysaccharide factor								
c286981_g1	551.5	8751.2	−3.8	3.43E-04	378	ACG60660.2	2.85E-52	Anti-lipopolysaccharide factor [*Macrobrachium rosenbergii*]
c210920_g1	428.2	3237.8	−2.8	1.73E-02	426	AFU61125.1	2.33E-44	Antilipopolysaccharide factor isoform 2 [*Fenneropenaeus chinensis*]
Tetraspanin								
c304292_g1	2692.6	324.6	2.9	3.56E-02	204	KFM58941.1	6.16E-11	Tetraspanin-11 [*Stegodyphus mimosarum*]
Crustin								
c231035_g1	1946.0	142.9	3.5	3.37E-02	426	ACU25382.1	1.08E-07	Crustin 1 [*Panulirus japonicus*]
c253822_g1	289.0	17.6	3.8	1.27E-02	276	ACU25385.1	9.46E-09	Crustin 4 [*Panulirus japonicus*]
c211503_g1	1903.7	84.0	4.2	1.21E-02	543	ACP40176.1	1.20E-28	Crustin Pm5 [*Penaeus monodon*]
LysM and putative peptidoglycan-binding domain-containing protein								
c322130_g1	0.4	40.3	−6.0	1.09E-03	864	XP_002730862.1	7.45E-18	LysM and putative peptidoglycan-binding domain-containing protein 3-like [*Saccoglossus kowalevskii*]
Heat shock protein								
c316776_g1	1269.8	88.8	3.7	1.65E-03	1938	AKB96227.1	0	Heat shock protein [*Cherax destructor*]
c323326_g1	1.1	111.5	−6.2	1.37E-05	1326	AET34915.1	5.62E-26	Heat shock protein 21 [*Macrobrachium rosenbergii*]
c309130_g2	2.9	35.5	−3.4	3.04E-02	1044	AET34915.1	2.41E-11	Heat shock protein 21 [*Macrobrachium rosenbergii*]
c322970_g1	51.8	2549.3	−5.3	1.19E-04	2787	AET34915.1	1.59E-54	Heat shock protein 21 [*Macrobrachium rosenbergii*]
c322631_g1	0.4	172.4	−7.8	2.22E-06	1095	AET34915.1	3.81E-16	Heat shock protein 21 [*Macrobrachium rosenbergii*]
c323430_g1	0.8	147.7	−6.7	1.25E-05	1697	AET34915.1	1.13E-18	Heat shock protein 21 [*Macrobrachium rosenbergii*]
c317639_g1	2.4	52.4	−4.2	9.31E-03	1212	AET34915.1	5.25E-24	Heat shock protein 21 [*Macrobrachium rosenbergii*]
c304475_g1	3	49.1	−3.8	3.07E-02	1662	AET34915.1	3.19E-44	Heat shock protein 21 [*Macrobrachium rosenbergii*]
c314609_g1	2.2	62.2	−4.4	1.60E-02	1437	AET34915.1	7.37E-12	Heat shock protein 21 [*Macrobrachium rosenbergii*]
c265283_g1	8.5	235.2	−4.5	3.33E-03	2266	ADM88040.1	0	Heat shock protein 90 [*Palaemon carinicauda*]
c270486_g2	0	18.6	−6.4	1.40E-02	1098	AAY67878.1	1.00E-106	Heat shock protein 90 [*Pseudourostyla cristata*]
c295139_g2	0	16.6	−6.3	1.90E-02	1565	XP_005971351.1	0	Heat shock 70 kDa protein [*Pantholops hodgsonii*]

^a^Padj, P adjusted value.

To validate the expression profiles, 24 DEGs were selected and subjected to qRT-PCR analysis. The results were in good agreement with those of RNA-Seq quantification, and the Pearson product-moment correlation coefficient between qRT-PCR-based and mRNA-Seq-based transcript abundance was 0.9586 (Fig. S3).

### DEGs involved in sulfur metabolism and detoxification

Unigenes encoding enzymes involved in sulfur oxidation were identified (Table 3). Two unigenes (c471705_g1 and c320892_g1) annotated as sulfite oxidase were upregulated in GR. One unigene (c52828_g1) annotated as SQR, which was reported to be a membrane-bound mitochondrial protein that catalyzes the formation of polysulfides using a quinone molecule^19, 20^, was upregulated GR (Table 3). The rhodanese (RHOD) domain-containing protein is a component of the mitochondrial H_2_S oxidation pathway, which catalyzes the transfer of sulfane sulfur from glutathione persulfide to sulfite generating thiosulfate^21^. Four unigenes belonging to the RHOD domain-containing protein were highly differentially expressed GR (Table 3). Of these genes, c317563_g2, which was upregulated in GR, was annotated as a thiosulfate sulfur transferase (TST); two other genes, c61012_g1 and c331935_g1, which were downregulated GR, were annotated as 3-mercaptopyruvate sulfur transferase (MPST).

Sulfotransferase catalyzes the conversion of sulfate to organic sulfur and is essential to the detoxification of numerous drugs and other xenobiotics^22^. A total of 13 unigenes annotated as sulfotransferases (SULTs) showed significantly higher expression levels in GR than in mGR (Table 3), including two heparan sulfate 2-O-sulfotransferases (c319825_g3 and c312613_g1), one galactosylceramide sulfotransferase (c294043_g1), two estrogen sulfotransferases (c310298_g1 and c32047_g1), one chondroitin 4-sulfotransferase (c275050_g1), and seven carbohydrate sulfotransferases (c321100_g1, c297891_g2, c321023_g2, c309372_g1, c281591_g1, c289839_g1, and c304186_g1) (Table 3). GO enrichment analysis also detected these genes and classified them as members of the significantly upregulated subcategory (Fig. 4).

### DEGs involved in anti-oxidative stress

**Figure 5:**
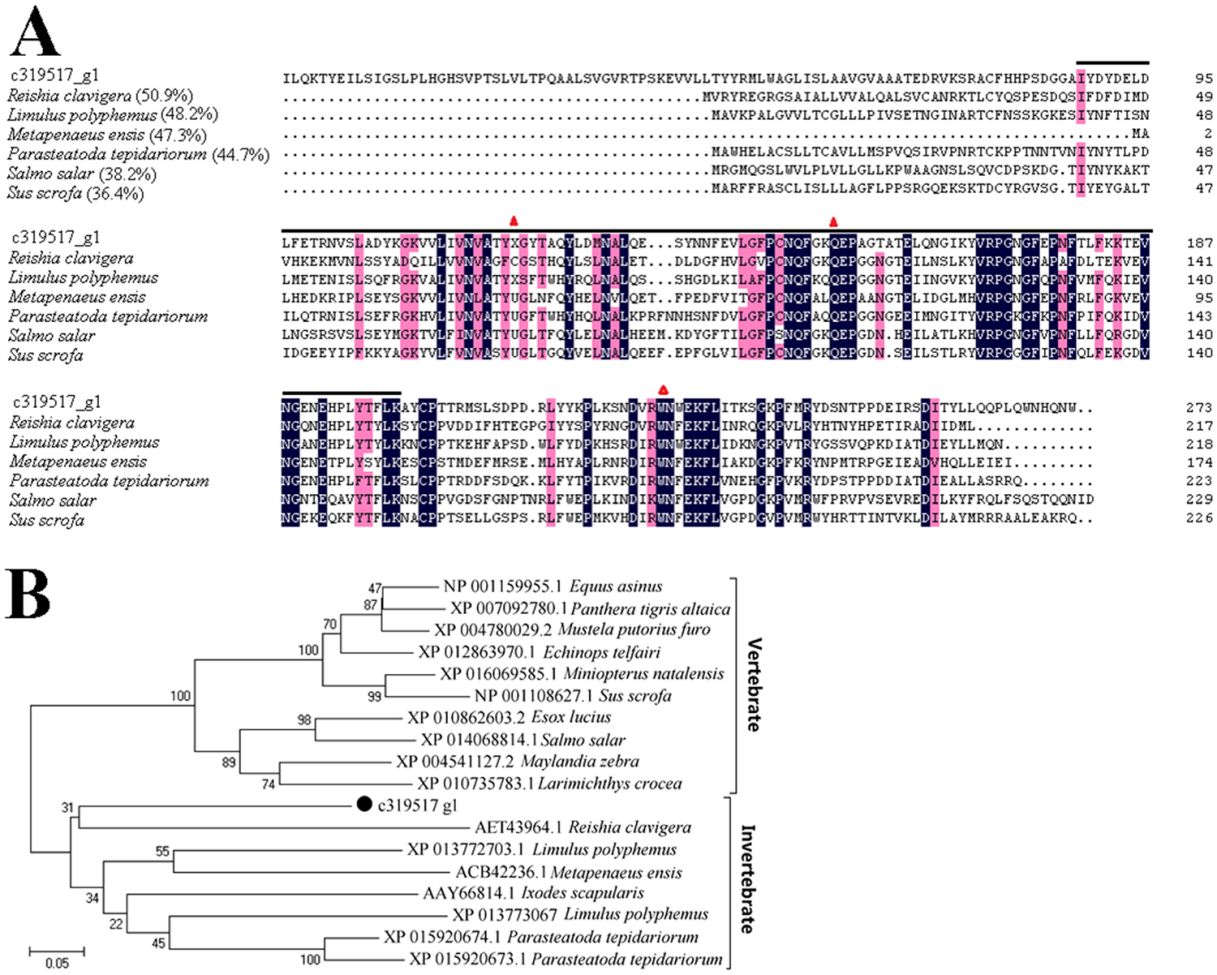
Sequence analysis of the glutathione peroxidase (GPx) (c319517_g1) from *Rimicaris* sp. (**A**) Sequence alignment of c319517_g1 and other GPx proteins from *Reishia clavigera*, *Limulus polyphemus*, *Metapenaeus ensis*, *Parasteatoda tepidariorum*, *Salmo salar*, and *Sus scrofa*. The numbers in brackets indicate overall sequence identities between c44577_g1 and the compared sequences; the consensus residues are in black; the residues that are ≥75% identical among the aligned sequences are in pink; the GSH peroxidase domain is indicated by the black line; the catalytic residues are represented by triangles. The GenBank Accession numbers of the aligned sequences are as follows: *Reishia clavigera* (AET43964), *Limulus polyphemus* (XP_013773067), *Metapenaeus ensis*, ACB42236; *Parasteatoda tepidariorum*, XP_015920674; *Salmo salar*, XP_014068814; *Sus scrofa*, NP_001108627. (**B**) Phylogenetic analysis of c319517_g1 (marked with “●”) and other GPx sequences with 1,000 bootstrap replications.

Antioxidant enzymes involved in defense against oxidative stress were identified in *Rimicaris* sp., including glutathione peroxidase (GPx), catalase (CAT), and superoxide dismutase (SOD)^23^. Four unigenes were annotated as copper/zinc superoxide dismutase (Cu/Zn SOD), two of which (c277048_g4 and c287566_g1) exhibited higher expression levels in GR than in mGR, and the other two (c293292_g1 and c518684_g1) exhibited lower expression levels in GR than in mGR (Table 3). One unigene, c319517_g1, is a GPx and highly expressed in both GR and mGR (49538.8 and 5728.6 reads, respectively); nevertheless, as confirmed by qRT-PCR, the expression level of this gene in GR was significantly higher than that in mGR (Table 3). c319517_g1 was highly homologous to the GPx of *R*. *clavigera* (50.9%), *Limulus polyphemus* (48.2%), *Metapenaeus ensis* (47.3%), and *Parasteatoda tepidariorum* (44.7%) (Fig. 5A). A relatively conserved GSH-peroxidase domain was detected in c319517_g1, and two of the three catalytic residues (Trp and Gln) that are conserved in vertebrates and invertebrates were also observed in c319517_g1 (Fig. 5A). Phylogenetic analysis classified c319517_g1 as a member of the invertebrate clade closely related to the GPx of *Reishia clavigera* (Fig. 5B).

**Figure 6:**
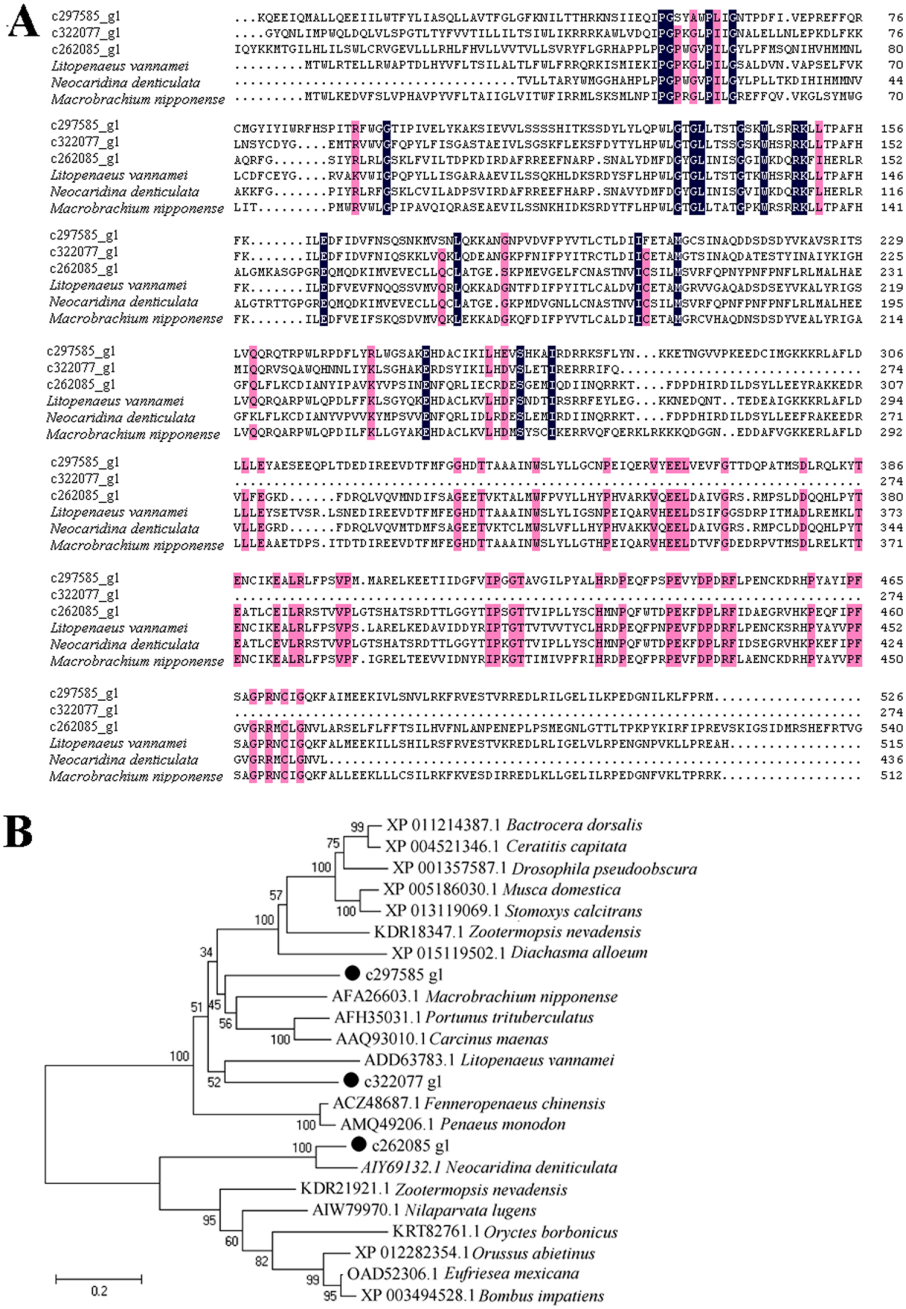
Sequence analysis of cytochrome P450 (CYP450) that was upregulated in *Rimicaris* sp. (**A**) Sequence alignment of CYP450 from *Rimicaris* sp., *Litopenaeus vannamei Neocaridina denticulate*, and *Macrobrachium nipponense*. The consensus residues are in black; the residues that are ≥75% identical among the aligned sequences are in pink. (**B**) Phylogenetic analysis of CYP450 by distance-based neighbor-joining method with 1,000 bootstrap replicates. The sequences marked with “●” are from this study. The GenBank Accession numbers of the aligned sequences are as follows: *Litopenaeus vannamei*, ADD63783.1; *Neocaridina denticulate*, AIY69132.1; and *Macrobrachium nipponense*, AFA26603.1.

Cytochrome P450 (CYP450) is a superfamily of hemeproteins that play a central role in oxidative metabolism and detoxication^24, 25^. Three unigenes (c322077_g1, c262085_g1, and c297585_g1) that best matched CYP450 were upregulated in GR (Table 3). These unigenes showed low to moderate sequence identities (13.1%-44.9%) with other known shrimp CYP450 (Fig. 6A). Phylogenetic analysis showed that c297585_g1 was clustered together with the CYP450 of the shrimp *Macrobrachium nipponense* and the crab *Portunus trituberculatus* and *Carcinus maenas*, c322077_g1 was clustered together with the CYP450 of the shrimp *Litopenaeus vannamei*, and c262085_g1 was clustered with the CYP450 of the shrimp *Neocaridina deniticulata* (Fig. 6B).

### DEGs involved in immunity

Ten unigenes annotated as C-type lectins were identified as DEGs between GR and mGR. Of these genes, two (c64342_g1 and c288904_g1) were upregulated and eight (c283074_g1, c316369_g1, c264506_g1, c312469_g4, c315345_g1, c292643_g1, c304905_g1, and c304072_g1) were downregulated in GR (Table 3). Three unigenes (c292762_g1, c273208_g1, and c276042_g1) encoding antimicrobial peptides (AMPs) and two unigenes (c286981_g1 and c210920_g1) encoding an anti-lipopolysaccharide factor were downregulated, whereas one unigene (c304292_g1) encoding tetraspanin and three unigenes (c231035_g1, c253822_g1, and c211503_g1) encoding crustin were upregulated in GR (Table 3). One unigene, c322130_g1, was homologous to LysM and the putative peptidoglycan-binding domain-containing protein (LYSMD). c322130_g1 possesses a highly conserved LysM domain and a less conserved transmembrane region; however, the amino acid sequence of c322130_g1 showed low levels of identity (≤ 24.7%) with the LYSMD sequences in NCBI (Fig. S4).

Heat shock proteins (Hsps) are classified into six families based on their molecular weights (kDa), i.e., Hsp100, Hsp90, Hsp70, Hsp60, Hsp40, and small Hsps^26^. In the present study, 12 Hsp-encoding unigenes were differentially expressed between GR and mGR. Of these genes, eight Hsp21, two Hsp90, and one Hsp70 were downregulated in GR, whereas one unigene annotated as Hsp70 was upregulated in GR (Table 3).

## Discussion

Shrimps have been discovered in various hydrothermal vents, however, the survival mechanism of these animals in the extreme conditions of hydrothermal systems is unknown. In this study, we employed the approach of transcriptome analysis to identify genes potentially associated with the local survival of the shrimp *Rimicaris* sp. from a hydrothermal vent in Desmos. Since *in situ* transcriptome along is unable to reveal which genes are specific and essential to local survival, comparative transcriptomes of contrasting conditions have to be conducted. For this reason, we compared the transcriptome profiles of the *Rimicaris* sp. directly from the field and the *Rimicaris* sp. that had been maintained in the laboratory. For the shrimp, the laboratory condition, which differed in many aspects from the field condition in the hydrothermal vent, was likely a “stress” condition. As a result, it is likely that some of the DEGs identified in our study were associated with stress response. However, the DEGs that were relatively highly expressed in the vent condition, such as those with known functions in the metabolism of hydrothermal vent-rich compounds, were probably required for survival in hydrothermal vent and may potentially be involved in adaptation.

In our results, 40.79 G clean bases were obtained from six individual shrimp and assembled into 488,739 unigenes. The number of unigenes detected in our study was much larger than that previously reported in the shallow sea shrimp *Litopenaeus vannamei* ^27^, which could be due to the significantly larger putative genome size (>10 pg) of *Rimicaris* compared to that of *L*. *vannamei* (2.5 pg)^28, 29^. However, only 128,938 (26.38%) transcripts had significant hits in known database, which is probably because (i) the information on shrimp genome sequence is very limited, and (ii) the transcripts of *Rimicaris* sp. are unique^29^. A total of 3,972 unigenes were differentially expressed between the GR and mGR samples, which suggests that changes in environmental condition significantly influence the expression profiles of various genes in *Rimicaris* sp. qRT-PCR analysis confirmed the expression patterns of 18 DEGs, indicating that the RNA-Seq data are highly reliable. The DEGs fell into a wide range of functional categories, including those likely associated with surviving in hydrothermal vent as discussed below.

### Sulfide exclusion and metabolism

A classic feature of hydrothermal vent is high concentrations of H_2_S^3^. Similarly, in the present study, we found a high concentration of H_2_S in the sampling site. H_2_S is a well-known toxic substance that can potentially harm living organisms, and organisms adapted to H_2_S-rich environments have evolved multiple strategies to deal with this toxicant^9^. One of these strategies is exclusion mechanism, which involves physical barriers that reduce the flux of H_2_S into the body^18^. In our study, we found that genes known to be involved in the formation of calcified cuticular structures of invertebrate animals^30, 31^, such as cuticular protein, chitin binding peritrophin, obstructor B, and laminin, were expressed in much higher levels in GR than in mGR, suggesting a possible existence of exclusion mechanism in *Rimicaris* sp. that minimizes H_2_S entry.

When H_2_S enters the body of the organism, the initial step of sulfide detoxification is the oxidation of H_2_S to thiosulfate by enzymes with sulfide oxidase activity^32, 33^. Previous studies on hydrothermal vent animals have revealed that the blood of *Riflia pachyptila* and *Calyptogena magnifica* possesses sulfide-binding proteins that protect sulfide-sensitive hemoglobins^34, 35^. However, such characteristics have not been found in shrimp. In the present study, unigenes annotated as mitochondrial sulfite oxidase were upregulated in GR. Given the importance of this enzyme in sulfide oxidation/detoxification, these unigenes may enable gills to act as a “peripheral defense” line to oxidize intracellular sulfide into sulfite as previously reported in cold seep mussels^12, 36^. Besides sulfite oxidase, SQR, another key enzyme that catalyzes the oxidation of sulfide to elemental sulfur^20, 37^, was also upregulated in GR. This observation coincides with the findings of previous reports showing that *Bathymodiolus platifrons* and other marine invertebrates inhabiting high-sulfide environments utilized sulfide oxidase and SQR to maintain endogenous H_2_S concentrations^12, 38^. RHOD domain-containing protein is also an important enzyme of H_2_S detoxification and plays a important role in cyanide-detoxification and anti-oxidative stress^20, 32, 39^. In the present study, a RHOD domain protein and a RHOD domain-containing protein annotated as TST were upregulated in GR, suggesting that these proteins may cooperate with sulfur oxidase to maintain sulfide homeostasis. In line with these results, two MPST genes were found to be downregulated in GR. MPST is a cytoplasmic and mitochondrial protein with two rhodanese domains and is a key enzyme in endogenous H_2_S generation^40, 41^. Taken together, these results suggest that the enhanced expression of H_2_S oxidizer and reduced expression of H_2_S generator facilitate the survival of *Rimicaris* sp. in the H_2_S-rich environment of hydrothermal vent.

When sulfate is assimilated or generated through sulfur oxidation by living organisms, it is reduced and converted to organic sulfur, which is an essential component of proteins^42^. In our study, numerous SULTs were significantly upregulated in GR. SULTs catalyze the transfer of a sulfonate (SO^3−^) group from the universal sulfate donor PAPS to an acceptor substrate, by which sulfate is reduced and converted to organic sulfur^42^. The upregulation of SULTs promote the ability of the organism to detoxify or excrete harmful compounds, including dietary xenobiotics, environmental pollutants, and drugs^42^. In addition, SULTs also participate in various critical biological processes such as immune response, virus infection, and wound repair^43^. The observation of a high number of upregulated SULTs in GR is indicative of the importance of SULTs in the adaptation of *Rimicaris* sp. to the toxic environment of hydrothermal vents.

### Anti-oxidative stress

CYP450 plays a role in H_2_S detoxication by catalyzing the oxidative metabolism of H_2_S^24, 25^. In our study, the expressions of three unigenes encoding CYP450 were upregulated in GR, suggesting that CYP450 may contribute to the protection of *Rimicaris* sp. by participating in H_2_S detoxification. However, during the detoxification process mediated by CYP450, a large amount of reactive oxygen species (ROS) is generated, which promotes oxidative stress^44, 45^. Oxidative stress in turn activates antioxidant cascade pathways, which include production of SOD, the first line of defense against oxidative stress^46^. In our study, four Cu/Zn SODs were identified as DEGs, two of which were upregulated in GR, whereas the other two were downregulated. Cu/Zn SOD has been reported to comprise 90% of the total SOD in mammalian cells^45, 47^. Cu/Zn SODs have also been reported in several crustaceans, including *Litopenaeus vanamei*, *Callinectes sapidus*, and *Scylla seratta* ^48^. Besides detoxification, Cu/Zn SOD proteins also participate in the generation of innate immune response^49, 50^. The differential expression patterns of Cu/Zn SODs observed in our study suggest that different isoforms of Cu/Zn SOD may have variable selectivity towards a range of challenges such as H_2_S, toxin, and pathogens.

In the antioxidant cascade pathways, H_2_O_2_ generated from SOD is simultaneously converted into water and oxygen by various scavenging enzymes such as ascorbate peroxidase, CAT, and GPx^23^. In the present study, GPx genes were upregulated, particularly in GR. GPx is known to be a peroxidase enzyme that catalyzes the reduction of hydrogen peroxide and hydrogen peroxide to water and oxygen, and thus is vital to cellular antioxidant defense^51^. The exceedingly high levels of GPx detected in GR indicate a potentially essential role of GPx in the antioxidant process of *Rimicaris* sp.

### Innate immune system

The innate immune system is essential for the survival of animals, particularly invertebrates that do not possess an adaptive immune system^52^. In the present study, various immune-relevant DEGs were identified, including typical pattern recognition receptors (PRRs) that detect and respond to pathogen-associated molecular patterns (PAMPs). Of the differentially expressed PRRs, 10 were annotated as lectins. Lectins have been found in almost all metazoans and are involved in immune recognition and phagocytosis^53–55^. Another type of PRRs identified in our study is a LYSMD homolog that contains a highly conserved LysM domain. LYSMD has been reported in plants, bacteria, and eukaryotes, and participates in specific host-bacteria recognition and degradation of bacterial cell walls^56, 57^. The identification of LYSMD in our study provides the first evidence that at least *Rimicaris* sp. possesses LYSMD as a potential PRR. Most DEG lectins as well as LYSMD were downregulated in GR, which may reflect the differences in the microbial communities encountered by the shrimp of GR and mGR groups. This hypothesis is in line with the findings of previous reports that the deep-sea hydrothermal vent shrimp *R*. *exoculata* hosts a dense community of epibiotic bacteria in its gill chambers, and that epibiotic bacteria affect the life cycle of the host shrimp^58–61^.

Besides PRRs, we also found DEGs encoding factors of the downstream immune response initiated by PRRs, notably AMPs. AMPs are effector proteins of the innate immune response and directly execute pathogen killing and clearance^62^. The production of AMPs is regulated by upstream immune signals such as those induced by PPR activation^63^. In the present study, we detected various types of AMPs, including classical AMPs (type 1 and 2 AMPs), crustins, and anti-lipopolysaccharide factor. Of the AMPs exhibiting differential expression patterns between GR and mGR, type 1 and 2 AMPs were downregulated, whereas crustins were upregulated, suggesting that different types of AMPs may possess different functions such as targeting different types of pathogens. This is likely, considering that compared to GR samples, mGR samples were collected from shrimp maintained under conditions different from that of GR in microbial environment.

Hsps play essential roles in heat shock tolerance and refolding denatured proteins^64^. They also respond to other stressors such as pathogen infection, oxidative stress, heavy metals, and other xenobiotics^65^. In the present study, most of the DEGs belonging to Hsp family were downregulated in GR, including Hsp70 and Hsp90, which play important roles in response to environmental pollutants, toxins, and bacterial and viral infections in both finfish and shrimp^66^. Eight small Hsps (Hsp21) were downregulated in GR. Currently, the function of Hsp21 in aquatic animals is unclear. In mammals, small Hsps block the aggregation of unfolded proteins, respond to oxidative stress, and display a cytoprotective function under stressful situations^67^. The observation that Hsps and other immune genes were downregulated in our study suggests that although the deep-sea hydrothermal vent is a harsh habitat from a conventional point of view, it is probably more suitable for local inhabitants such as *Rimicaris* sp. that have evolved adaption mechanisms to thrive in the specific environment.

### Conclusions

In this report we presented for the first time a transcriptome database for the deep-sea hydrothermal vent shrimp *Rimicaris* sp., hence providing a comprehensive resource for molecular studies of this species. Through comparative analysis, a large amount of genes differentially expressed between *in situ* and laboratory conditions were identified. Since for deep-sea hydrothermal vent animals, it is very difficult to perform experiments to distinguish between genes of stress response and adaptation, it cannot be ruled out that some of the DEGs identified in our study were expressed temporarily as a reaction to stress; however, there were DEGs in our study, especially those with predicted functions in biological processes (such as sulfur metabolism and anti-oxidation) involving compounds characteristically enriched in hydrothermal systems, that were most likely essential to hydrothermal vent survival and may potentially contribute to adaptation as well. Given the present lack of knowledge with respect to the evolutionary adaptation mechanism of deep-sea animals, our results provide at least potential gene targets and a clue to the molecular basis of adaptation in *Rimicaris* sp. and will serve as important reference data for future studies of deep-sea shrimp.

## Materials and Methods

### Ethics statement

The samples used in this study were collected in June, 2015 during the cruise conducted by the scientific research vessel KEXUE in Manus Basin. The cruise was approved by Chinese Ministry of Foreign Affairs and with permit from the relevant country. This study did not involve any endangered or protected species. Live animal research was approved by the Ethics Committee of the Institute of Oceanology, Chinese Academy of Sciences.

### Sample collection and manipulation

The sampling site is located in the southeast of the Desmos Manus hydrothermal field in a new hydrothermal vent with white smokers that was discovered during a cruise to the Manus back-arc basin that was conducted by the scientific research vessel “KEXUE” in June 2015 (Fig. 1). Dense populations of *Rimicaris* shrimp in close proximity to the vent (151°52.63′E, 3°42.27′S, ~1883 m) were discovered and collected by the ROV “Fa Xian” (Fig. 1). The *in situ* environmental conditions were obtained by using the sensors of the ROV. The temperature of the sampling site was between 4 °C and 20 °C, the dissolved oxygen was ~3.6 mg/L, and the dissolved carbon dioxide was ~657.0 mg/L. A high concentration of hydrogen sulfide (>10 mmol) was detected by a deep ocean *Raman in*-*situ* spectrometer (Raman insertion probe). For convenience of comparison, the shrimp were named as the DM (Desmos Manus) isolate. Once on board, six live female shrimps with an average length of 4 ± 0.2 cm were selected, three of the individuals were immediately dissected, and the gills were flash-frozen in liquid nitrogen and stored at −80 °C for RNA extraction. These gill samples were named GR (Gill of *Rimicaris* sp.) 1, GR2, and GR3. The other three selected individuals were maintained by being placed in a PVC tank containing 5 L of seawater that had been filtered through 0.22-μm filters. The tank was placed in a cold room with a constant temperature of 10 ± 1 °C and renewed every day with freshly filtered seawater, in which the dissolved oxygen and carbon dioxide of the seawater were ~7.0 mg/L and ~402.0 mg/L, respectively, and no hydrogen sulfide could be detected. After 10 days of maintenance, the shrimp were sacrificed, and the gills (named mGR1, mGR2 and mGR3) were collected under aseptic conditions. The gill samples were flash-frozen and stored as earlier described.

### Phylogenetic analysis

Shrimp were genotyped by sequencing the genes of mitochondrial cytochrome c oxidase I (COI) and 16S rRNA using previously reported primers^68, 69^. Briefly, total RNA was extracted from the shrimp by using an EZNA total RNA kit (Omega Bio-tek, Doraville, USA), and cDNA synthesis was performed as previously reported^70^. Partial COI and 16S rRNA genes were amplified using primer pairs COIL1490/COIH2198 and 16S AR/16S BR, respectively. The PCR products were purified and sequenced, and the sequences were deposited to the National Center for Biotechnology Information (NCBI) as Accession numbers KX228390 (COI) and KX228389 (16S). The gene sequences were subjected to BLAST analysis. Multiple sequence alignment was performed with Megalign (DNASTAR) by using the Clustal V method. Phylogenetic analysis was performed with the neighbor-joining method with 1,000 bootstrap replicates using MEGA 5.0.

### RNA extraction and library preparation

Approximately 50 mg of gills from each shrimp were used for RNA extraction with a HP total RNA kit (Omega Bio-tek). DNA was removed from the RNA sample with a RNase-free DNase set (Omega Bio-tek). RNA quality was examined by 1% agarose gel electrophoresis. RNA concentration was measured using a Qubit® RNA assay kit in a Qubit® 2.0 Fluorometer (Life Technologies, CA, USA). RNA integrity was assessed by using a RNA Nano 6000 assay kit (Agilent Technologies, CA, USA).

A total of 1.5 µg of RNA per sample was used as input material for RNA library preparation. Sequencing libraries were generated using NEBNext® Ultra™ RNA Library Prep Kit for Illumina® (NEB, USA), following the manufacturer’s recommendations, and index codes were added to attribute sequences to each sample. Briefly, mRNA was purified from total RNA using polyT oligo-attached magnetic beads. After fragmentation, double-stranded cDNA was synthesized a using random hexamer primer and M-MuLV reverse transcriptase (Superscript II, Life Technologies, CA, USA). The synthesized cDNA was subjected to end-repair, phosphorylation, 3′ adenylation, and ligation of adapters. To select cDNA fragments of 150~200 bp in length, the library fragments were purified with an AMPure XP system (Beckman Coulter, Beverly, USA). Then, 3 µL of the USER enzyme (NEB, USA) was incubated with size-selected, adaptor-ligated cDNA at 37 °C for 15 min, followed by 5 min at 95 °C before PCR. PCR was performed with a Phusion High-Fidelity DNA polymerase, universal PCR primers, and an index (X) primer. The PCR products were purified (AMPure XP system), and library quality was assessed on an Agilent Bioanalyzer 2100 system. The clustering of the index-coded samples was performed on a cBot Cluster generation system using a TruSeq PE cluster kit v3-cBot-HS (Illumina). After cluster generation, the library preparations were sequenced on an Illumina Hiseq 4000 platform at Novogene (Beijing, China), and 150-bp paired-end reads were generated.

### Illumina sequencing, assembly, and annotation

To obtain clean reads, the raw reads were cleaned by removing reads with adaptors, poly-N of >10%, and low-quality reads (the percentage of the low-quality bases of Q ≤ 20 being >50% in a read). Transcriptome assembly was performed by using Trinity (r20140413p1) as described for *de novo* transcriptome assembly without a reference genome^71, 72^, with min_kmer_cov set to 2 by default and all other parameters set default. The longest transcript of each transcription set was defined as the ‘unigene’ for further functional annotation. All assembled unigenes of the samples were analyzed against public databases, including NCBI non-redundant protein (Nr), NCBI nucleotide (Nt), and SwissProt, using BLAST with an E-value of 10^−5^, the eukaryotic orthologous Eukaryotic Orthologous Groups (KOG) database with E-value of 10^−3^, and the Kyoto Encyclopedia of Genes and Genomes (KEGG) database with an E-value of 10^−10^. PFAM protein family alignments were performed using the HMMER (v3.0) package with an E-value of 10^−2^, GO classification was conducted using Blast2GO (v2.5)^73, 74^ with an E-value of 10^−6^, and KEGG classification was performed using KASS (vr140224)^75^ and the KEGG Automatic Annotation Server.

### Identification of DEGs

Gene expression levels in each sample were estimated by mapping clean reads to the Trinity unigenes that were assembled by RSEM^76^. The abundance of all genes was normalized and calculated using uniquely mapped reads by the expected number of Fragments Per Kilobase of unigene sequence per million (FKPM) base pairs sequenced method, which takes into account the influence of both sequencing depth and gene length on read count^77^. Read count was normalized using the TMM method^78^. Differential expression analysis of all samples was performed using the DESeq R package (1.10.1) based on the negative binomial distribution^79^. The resulting p-values were adjusted using the Benjamini and Hochberg’s approach, and the p-adjusted value (padj < 0.05) was set as the threshold for significant differential expression^80^. The identified DEGs were then implemented for GO enrichment analyses. GO enrichment analysis of DEGs was performed using GOseq R packages based on the Wallenius non-central hypergeometric distribution, which can adjust for gene length bias in DEGs^81^.

### Validation of DEGs by quantitative real-time reverse transcription-PCR (qRT-PCR)

To validate the DEGs obtained by RNA sequencing, qRT-PCR of 18 DEGs was conducted using the total RNA that was used for RNA sequencing. The primer sequences used in this study are listed in Table S1. RNA was treated with RNase-free DNaseI (TaKaRa, Dalian, China). One microgram of RNA was used for cDNA synthesis with Superscript II reverse transcriptase (Invitrogen, Carlsbad, CA, USA). qRT-PCR was performed in an Eppendorf Mastercycler (Eppendorf, Hamburg, Germany) using a SYBR ExScript qRT-PCR kit (Takara, Dalian, China) as reported elsewhere^82^. Melting curve analysis of the amplification products was performed at the end of each PCR to confirm that only one PCR product was amplified and detected. The expression level of the target genes was analyzed using the comparative threshold cycle method (2^−ΔΔCT^) with *β*-*actin* as internal control^83^.

### Statistical analysis

All experiments were performed three times. Statistical analyses were performed using the SPSS 17.0 software (SPSS Inc., Chicago, IL, USA). Data were analyzed with analysis of variance (ANOVA), and statistical significance was defined as *P* < 0.05.

## Electronic supplementary material

Supplementary information
