# Supplementary material for: Comparative transcriptome analysis of Rimicaris sp. reveals novel molecular features associated with survival in deep-sea hydrothermal vent
Source: Sci Rep. 2017 May 17;7:2000. doi: 10.1038/s41598-017-02073-9 (PMC5435735; doi:10.1038/s41598-017-02073-9)

Comparative transcriptome analysis of *Rimicaris* sp. reveals novel molecular features associated with adaptation to deep-sea hydrothermal vent

Jian Zhang[a,b,d], Qing-lei Sun[a,b], Zhen-dong Luan[b,c,d], Chao Lian[c], Li Sun[a,b,]*

[a]*Key Laboratory of Experimental Marine Biology, Institute of Oceanology, Chinese Academy of Sciences, Qingdao, 266071, China*
[b]*Laboratory for Marine Biology and Biotechnology, Qingdao National Laboratory for Marine Science and Technology, Qingdao, China*
[c]*Key Laboratory of Marine Geology and Environment, Institute of Oceanology, Chinese Academy of Sciences, Qingdao 266071, China*
[d]*Deep sea research center, Institute of Oceanology, Chinese Academy of Sciences, Qingdao 266071, China*

*To whom correspondence should be addressed

Mailing address: Li Sun
Institute of Oceanology
Chinese Academy of Sciences
7 Nanhai Road
Qingdao 266071, China
Phone: 86-532-82898829
Email: lsun@qdio.ac.cn

Running title: Comparative transcriptome analysis of *Rimicaris* sp. from hydrothermal vent

Supplementary tables

Table S1. qRT-PCR primers used in this study.

Transcript ID	Transcript name	Forward primer	Tm (℃)	Reverse Primer	Tm (℃)
c323326_g1	Heat shock protein 21	GTGAAAGTGAAAGCAAAGGAAATGG	57.0	GCCAACAGCAAGTGAGTCATTC	57.1
c265283_g1	Heat shock protein 90	CGATGCTGACAAGAACGACAAG	56.9	ATCAATGCCAAGACCGAGACC	56.8
c295139_g2	Heat shock 70 kDa protein	TTGGACGAGCACAAGGAGAAG	56.7	TCATGGAGGCAGCACTCAATG	57.1
c231035_g1	Crustin 1	GCGTAGCCATGTGCCAGAG	57.2	CGTTATTGTGACCGTTGCCTAG	56.4
c253822_g1	Crustin 4	GTGCGATCATATTGTCCGTCTTAC	57.0	TCCGAAATGTTGAGGTGATTTGC	56.9
c52828_g1	Sulfide:quinone oxidoreductase	AAACTCTTCAGCATGACGATTTCC	56.9	CCAGTAACCAAAGGACATGAAGTG	56.9
c235883_g1	Rhodanese domain protein	AGCAGTTCAGAGTGGCAATCC	57.0	GCCCAACAAACCCTCCTACC	56.7
c317563_g2	Thiosulfate sulfurtransferase/rhodanese-like	ATTTTCATTCCCACTGACTGAGATG	56.6	ATTATTTCGCCTGCCTTTCTTCC	56.5
c61012_g1	Thiosulfate/3-mercaptopyruvate sulfurtransferase	ACGCTTCACAGTCACTCTTACC	57.0	CCACTCAGTCCAAGATCCATCATAG	57.7
c318457_g1	Sulfite oxidase	ATGGACTGGACACGGTACTATTAAG	57.2	AGAATCATCATACGCACGACTTTG	56.9
c137930_g1	Metallothionein	AACTGTGGCTGCGGTTCTAG	56.5	CCCATCTCAGATCCCTCAAAGTG	57.3
c292762_g1	Antimicrobial peptide type 1 precursor	CCTCAAATCCTTCCAATCCCTTATG	56.7	GTCCCTTTCTGATGCCATTTCTG	56.7
c273208_g2	Antimicrobial peptide type 2 precursor IIc	TGTGAGAACGCCAACCAACC	57.3	CGTAGCAGCACTTGTCAACAC	56.6
c210920_g1	Anti-lipopolysaccharide factor isoform 6	TTGTACTTCAGCGGCAATATGTG	56.5	TGGCTTCGTCTTCCGTGATAAG	57.1
c312469_g4	C-type lectin 1	ACGAAGGAGATTGGAAGTGGATG	57.2	GAAGAAATGCTGGTGGGAAAGAC	56.9
c64342_g1	C-type lectin 4	CCTCAGTGTCTTGGGATGGTTC	57.3	ACTCGCTGGTCTGGTTAATGTC	57.0
c322130_g1	LysM and putative peptidoglycan-binding	AGCAGTTGTAGTGAGTATGAAAGTG	56.3	TCATTCAGAGCCGCAGCAG	56.4
c319517_g1	Glutathione peroxidase	GACAGAAGATCGCGTCAAATCGC	56.5	AGTTGTTGTAGGATTCTTGAAG	56.4
c277048_g4	Copper/zinc superoxide dismutase	ACTGAGCTATCTCAAGACTGTGGA	57.2	AGTTACTTTGGCTCCACATTTCTTTG	57.0
c297585_g1	Cytochrome P450	TACTCCGGACTTTATCGTCGAGC	56.7	GTAGATGTAAGAAGTCCTGTGC	57.2
c294043_g1	Galactosylceramide sulfotransferase	ACTACACAGGAACAGCGATAGTGT	57.5	CTCCGAAGTAGTTGGAGGTA	57.2

c310298_g1	Estrogen sulfotransferase	ATGCGCGAGTACCATTCCTTGAATG	56.9	ATGGAAGGAGACGACCACATCCT	56.6
c275050_g1	Chondroitin 4-sulfotransferase	AGTCCGAGACGATATGAAGGTC	56.7	GAGGCATGCGTGTGAGCATTCG	56.7
c321100_g1	Carbohydrate sulfotransferase 5	GAGGACTCGCCAAACTGTGCG	57.3	ATCATCTGCTGGGTGCCTATAAAC	57.1
c310092_g1	Beta-actin	CAGGAATCGCCGACAGGATG	57.1	TTTGTTGGAAGGTGGATAGAGAGG	57.0

Supplementary figures

Figure S1. Sequence length distribution of the transcripts assembled from Illumina reads. (A) Length information of the assembled transcripts. (B) Length interval distribution of transcripts.

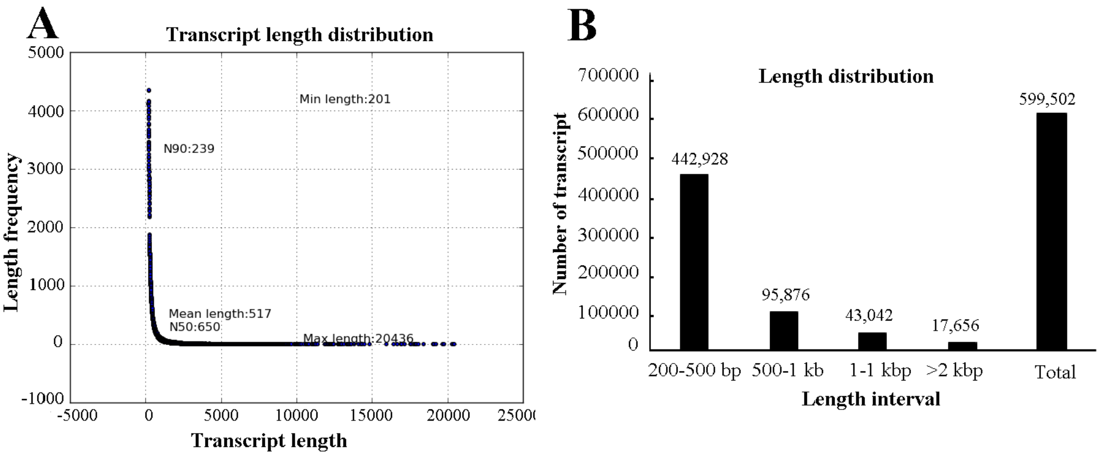

Figure S2. Characteristics of the homology search of the assembled unigenes against the Nr database. (**A**) Species distribution of all homologues of the assembled unigenes with an *E*-value of at least 10^{-5}. (**B**) *E*-value distribution of each unigene with a cut-off *E*-value of 10^{-5}.

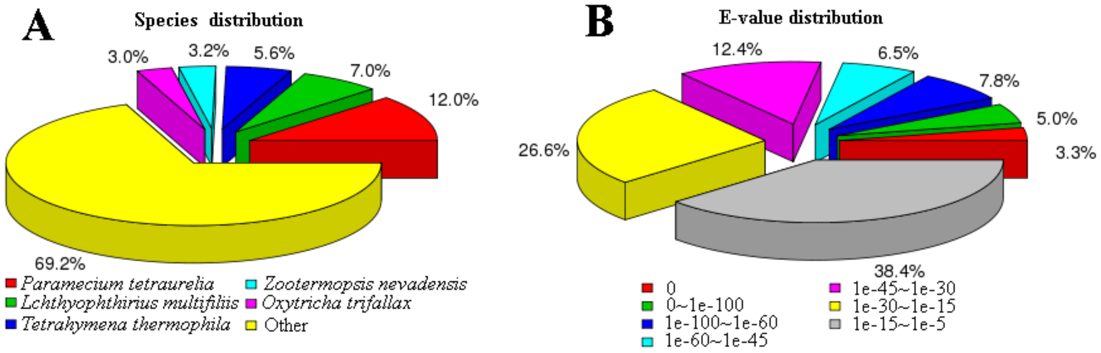

Fig. S3. RNAseq quantification versus quantitative real time RT-PCR (qRT-PCR) analysis. The qRT-PCR results of 24 DEGs were plotted against RNAseq quantification data. The Pearson product-moment correlation coefficient (R^2) of each plot was shown as an indication of positive linear correlation between the two methods.

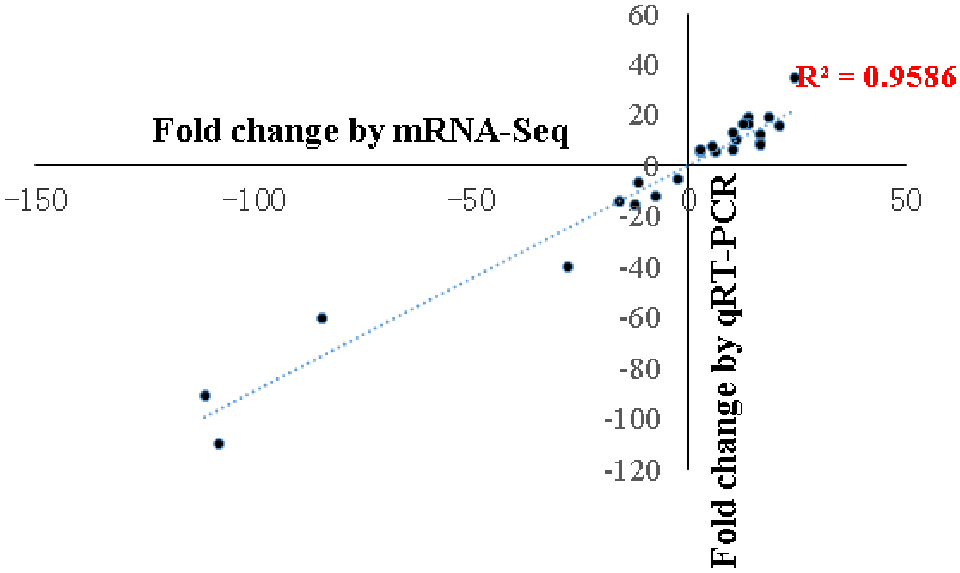

Fig. S4. Sequence analysis of LysM and putative peptidoglycan-binding domain-containing protein (LYSMD). Numbers in brackets indicate overall sequence identities between c322130_g1 and the compared sequences. The LysM domain and transmembrane region are indicated by green box and red line, respectively. The GenBank accession numbers of the aligned sequences are as follows: *Priapulus caudatus*, XP_014671252; *Saccoglossus kowalevskii*, XP_002730862; *Crassostrea gigas*, XP_011450399; *Xenopus tropicalis*, NP_001017308.

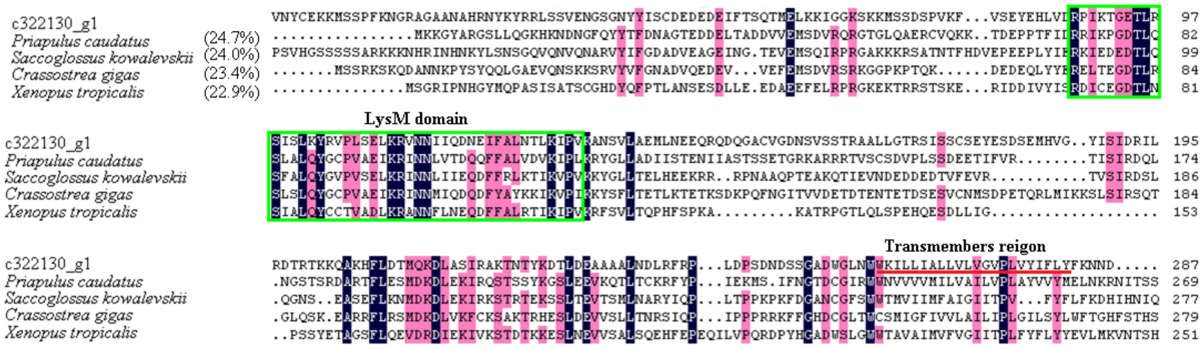

Supplement: Supplementary file 1 — Supplementary information [file 41598_2017_2073_MOESM1_ESM.pdf]
